# Sublethal and lethal toxicity assessment of lanthanum and gadolinium to *Daphnia magna* in a 7-day test method

**DOI:** 10.1007/s11356-024-35854-7

**Published:** 2025-01-10

**Authors:** Edith Guadalupe Padilla Suarez, Marion Revel, Giovanni Libralato, Marco Guida, Susanne Heise

**Affiliations:** 1 Biology Department, University of Naples Federico II, Via Cinthia 21, 80126 Naples, Italy; 2 Life Sciences, Hamburg University of Applied Science, Ulmenliet 20, 21033 Hamburg, Germany; 3 University of the West of Scotland, Paisley, PA1 2BE UK

**Keywords:** Rare earth elements, Lanthanoids, Non-standard testing, Crustaceans, Long-term exposure

## Abstract

The use of rare earth elements has increased in recent years, leading to a rise in environmental concentrations. Despite the growth in number of studies regarding toxicity, knowledge gaps remain. For *Daphnia magna*, standardized test methods involve exposure periods of either 48 h or 21 days to assess toxicological effects. In this study, the exposure period was adjusted to 7 days to evaluate sublethal endpoints not measurable in 48-h tests. Additionally, this approach enabled us to obtain results within a shorter time frame than that required for 21-day tests. This study focused on the individual toxicity of lanthanum (La) and gadolinium (Gd) to *Daphnia magna*. We assessed mortality, feeding rate, somatic growth, and maturity under static conditions, modifying the media by adding MOPS buffer to maintain an initial pH of 6.8 and providing *Raphidocelis subcapitata* as a daily food source. Results showed that the solubility of La decreased considerably, with the highest recovery rate dropping from 133.33% at the start to 32.73% by the end of the 7-day exposure period. In contrast, Gd solubility remained stable, with a recovery rate of 86.88% at the start and 81.30% at the end of the test. Daily lethal concentrations (LC_x_) were calculated, revealing LC_10_ values on the first day, LC_20_ on the second day, and LC_50_ by the third day for La and the second day for Gd. By the test’s end, the LC_10_, LC_20_, and LC_50_ values were 30.40, 78.96, and 403.67 µg L^−1^ for La, and 10.67, 33.73, and 241.28 µg L^−1^ for Gd. For the sublethal endpoints, maturity was the most sensitive endpoint with the EC_20_ and EC_10_ corresponding to 0.79 and 0.26 µg L^−1^ for La and 0.39 and 0.14 µg L^−1^ for Gd. Gd had a higher toxicity in all endpoints assessed. While a thorough comparison to existing literature remains challenging due to variations in endpoints assessed, the methodology employed in this study yielded a range of informative results. This approach provides a useful range-finding test for *Daphnia magna* toxicity assessments, particularly for preliminary screening, and may complement standardized methodologies.

**Supplementary Information:**

The online version contains supplementary material available at 10.1007/s11356-024-35854-7.

## Introduction

Rare earth elements (REEs), including the lanthanoids, yttrium (Y), and scandium (Sc), have gained increasing environmental relevance due to their widespread use in technology, medicine, and agriculture (Opare et al. 2021; Zhou et al. 2017). Though naturally occurring in trace amounts, rising REE concentrations in the environment, particularly in aquatic systems, pose potential ecotoxicological risks to organisms (Gwenzi et al. 2021; Haxel 2002; Pagano et al. 2015).

The unique physicochemical properties of REEs are crucial to understanding their bioavailability and ecotoxicological impacts. REEs can be categorized into light rare-earth elements (LREEs) and heavy rare-earth elements (HREEs) (Hower et al. 2016), with their ionic radii of REEs progressively decrease across the series. The contraction of lanthanide radii is associated with increasing charge density, contributing to the greater complexation stability of HREEs compared to LREEs (Cantrell and Byrne 1987; Grimes and Nash 2014; Weltje et al. 2002b). Additionally, lanthanide contraction facilitates the substitution of REEs for elements with similar atomic radii, such as calcium (Hovey et al. 2023). REEs can adopt a coordination number of 8 or 9, allowing them to form stable complexes with compatible ligands (Hovey et al. 2023).

External factors significantly influence the speciation and bioavailability of REEs, as well as their associated toxicity. For example, in water with pH below 7–8, REEs primarily exist as free ions (Takeno 2005). The presence of inorganic ligands such as phosphate, carbonate, sulfate, and chloride, along with organic colloids like humic acids and natural organic matter, can alter their solubility and sorption to surfaces (Cantrell and Byrne 1987; Davranche et al. 2008; Marsac et al. 2013; Millero 1992). However, a comprehensive understanding of how changes in exposure conditions affect toxicity endpoints remains elusive, leading to variability in effect thresholds across studies (Gonzalez et al. 2014; Herrmann et al. 2016).

Recent studies have established EC_50_ values for multiple REEs using 48-h acute tests with the freshwater model species *Daphnia magna*, where immobilization serves as the endpoint (Blinova et al. 2018; Lachaux et al. 2022; Vignati et al. 2024). Additionally, research involving *Daphnia similis* has contributed to this understanding (Egler et al. 2023). These investigations reveal that test medium composition can significantly affect effective concentrations, while solubility differences complicate toxicity comparisons across different REEs, even within the same study. Further variations in results are influenced by whether nominal, measured, or modeled concentrations are used, particularly regarding free ion concentrations.

Moreover, studies have investigated longer exposure periods for *Daphnia magna* to REEs to assess sub-lethal effects in long-term exposures (Blinova et al. 2018, 2022; Lürling and Tolman 2010; Salvatore et al. 2024) and multigenerational exposures (Galdiero et al. 2019). Such studies provide valuable insights into life history traits by examining endpoints that reflect the organisms’ responses over extended periods, allowing for the use of lower, more environmentally relevant concentrations and conditions. However, these studies can be time-consuming and logistically impractical (Lazorchak et al. 2009).

Building on these concepts, this study investigates both lethal and sub-lethal effects of lanthanum (La) and gadolinium (Gd) on *Daphnia magna* over a 7-day exposure period. Given La and Gd’s distinct positions in the REE series, they offer insight into how variations in physicochemical properties impact toxicity outcomes. By adapting OECD guidelines for acute and reproductive toxicity testing (OECD 2004, 2012), we aim to capture key endpoints such as mortality, feeding rate, maturity, and somatic growth. This approach addresses previous limitations in acute toxicity testing and long-term reproduction studies, contributing to the understanding of how REEs’ physicochemical properties influence their ecological impact. Our findings aim to enhance understanding of REE behavior in freshwater environments and their toxicity under laboratory conditions with a higher level of complexity that could be more environmentally relevant.

## Materials and methods

### Test organisms

*Daphnia magna* (Umweltbundesamt, Germany) is kept in a permanent culture in the work group of Applied Aquatic Toxicology at the University of Applied Sciences of Hamburg (HAW). The culture is maintained in a climate-controlled chamber in Elendt M4-media (OECD 211) at 21 ± 1 ℃ at a 16:8 h dark:light photoperiod and fed daily with the green microalgae *Chlorella vulgaris.*

### Chemicals

Experiments were conducted using commercially available solutions containing 1 g L^−1^ of GdNO_3_ (N9300118, PerkinElmer, Massachusetts, U.S.A.) and LaNO_3_ (N9300127, PerkinElmer). The exposure concentrations were prepared in a M4 media (Elendt 1990; OECD 2012) with the following modifications: to avoid chelation, the addition of EDTA was omitted from the solution, and 3-(*N*-Morpholino)-propane sulphonic acid (MOPS) buffer solution purchased as MOPS 99.5% (6979.3, Carl Roth, Karlsruhe, Germany) adjusted to pH 7 was added to maintain a neutral pH. The effects of MOPS on *Daphnia magna* have been previously studied (De Schamphelaere et al. 2004), demonstrating no significant impacts on long-term tests, which supports its use as a buffering agent in our experiments. The nominal REE concentrations corresponded to a negative control of 0 µg L^−1^, five concentrations of exposure: 500, 1000, 1750, 2500, and 5000 µg L^−1^ for La, and two additional concentrations for Gd due to the higher mortality induced, corresponding to: 500, 750, 1000, 1250, 1750, 2500, and 5000 µg L^−1^. Solutions were prepared immediately before use, and the pH was adjusted with 1M HCl or NaOH as necessary to achieve a pH of 6.8 (± 0.2).

### Toxicity tests

The exposure methods performed for this test were based on the OECD guidelines, test no. 202 for acute effects (OECD 2004), and test no. 211 for reproduction effects (OECD 2012). The duration of the test was adjusted to perform a test with a duration of 7 days under static conditions (no water exchange).

For each concentration, three PET plastic vessels with five individuals of < 24 h of age in 50 ml of test medium were prepared. PET vessels were selected alternatively to glass to avoid the adsorption into the glass (Weltje et al. 2002a).

To ensure a minimum of four replicates per day for daily mortality across all concentrations, the tests were conducted multiple times, with La being tested 11 times and Gd 14 times. Light and temperature conditions were maintained the same as those for the culture. Daphnids were fed daily with the microalgae *Raphidocelis subcapitata* (8 × 10^6^ cells day^−1^). The control’s test validity criteria for mortality were set at below 20%. The assessed endpoints included mortality, somatic growth, feeding rate, and sexual maturity (reproduction).

A daily assessment of the daphnids was performed to record the mortality of the daphnids, as well as the number of daphnids reaching sexual maturity. A daphnid was considered to have reached sexual maturity with the first appearance of eggs. At the end of the test, live daphnids were photographed under a binocular with a Moticam 5 (Motic, Hong Kong, China), and the size was measured (distance from the eye to the end of the tail) with the software ImageJ (Schneider et al. 2012).

The cell concentration of *R. subcapitata* was determined at the end of the test by fluorescence measurement (Tecan Infinite F200, TECAN Trading AG, Switzerland; measurement at 465 nm excitation and 680 nm emission). Along with the increase of algal cells caused by the daily addition of food, it was assumed that a slight growth of algae under exposure conditions also occurred. Therefore, an additional test of 72 h of *R. subcapitata* exposed to all tested concentrations was performed to estimate a growth constant (Supplementary Information). With the known number of algae added and the assumed growth, a theoretical final concentration was then used to subtract the measured concentration to calculate the cells consumed. Moreover, the number of alive daphnids per day was considered to calculate the feeding rate (cells consumed per daphnid per day).

### Chemical analysis

Samples for analysis were taken from one of the multiple tests performed at the start (*t* = 0) and at the end of the test (*t* = 7 days) and filtrated with a 2 µm cellulose nitrate filter (Whatman, 7182–004) to remove any metal precipitates and traces of the algae used for food. After filtration, HNO_3_ (4989.1, Carl Roth) was added to reach a concentration of 2%, and samples were maintained in polyethylene containers and stored at 4 ℃ until analysis. The concentrations tested were analyzed in three replicates using Inductively Coupled Plasma Mass Spectrometry (iCAP RQplus ICP-MS, Thermo Fisher) at the Chrono-environment laboratory (UMR CNRS 6249). The percentage of recovery was considered accurate within the recovery range between 80 and 120%.

### Data analysis

Data were analyzed with R Studio for Windows (Version: 2022.12.0 + 353) (Posit team 2024). To estimate the effective concentration of the endpoints tested, data were fit into dose–response models using the extension package “drc” (Ritz et al. 2015). For both lethal and sublethal endpoints, the effect concentrations were estimated using the measured concentrations at *t* = 7 d to address the worst-case scenario in this set-up.

For the lethal endpoint, the daily lethal concentrations causing 50, 20, and 10% of mortality (LC_50_, LC_20_, and LC_10_) were determined by fitting the data into a binomial log-logistic model with two parameters. For the sublethal endpoints of feeding, somatic growth, and reproduction, the effective concentrations causing 10 and 20% of the effect (EC_10_ and EC_20_) were estimated. Data of the feeding and somatic growth were fit into four parameters Weibull and log-logistic models, while for the reproduction, data were fit into a two parameters log-logistic model, selected based on the lowest Akaike’s information criterion.

Further analysis was performed to compare the response of the different concentrations of exposure by a Tukey’s test, with the significance level set at 5%, using the extension package “multcomp” (Hothorn et al. 2008). Letter coding for levels of significance (e.g., “a”, “b”, “c”) indicates statistically significant differences between groups, with letters that are not shared between groups denoting significant differences. For the reproduction endpoint, the percentage of matured daphnids was calculated based on the number of daphnids at the start of the test and pooled from all tests performed. For the feeding and somatic growth, the effect of each treatment was corrected with their respective control, and data were later pooled for analysis. Information regarding *p*-values provided in the Supplementary Information.

The relationship between the feeding rate and size of alive daphnids at the end of the test was further examined by linear regression.

## Results

### Measured concentrations

The nominal and measured concentrations at *t* = 0 and *t* = 7 of La and Gd are displayed in Table 1. For the measured concentrations at *t* = 0, those of La were closer to the nominal and overall higher than those of Gd. At *t* = 7, a different trend is observed, where La had lower concentrations compared to Gd. Additionally, for both elements, at the two sampling times, the recovery rates increased with increasing concentration (except the highest concentrations of La at *t* = 7).

**Table 1:** Mean measured concentrations (µg L^−1^) and recovery rate (%) of La and Gd at the start of the test (*t* = 0) and after 7 days of exposure (*t* = 7)

Nominal concentrations (µg L^−1^)	Measured concentrations *t* = 0 (µg L^−1^)		Recovery rates (%)		Measured concentrations *t* = 7 (µg L^−1^)		Recovery rates (%)	
	La	Gd	La	Gd	La	Gd	La	Gd
0.0	0.00	0.00	-	-	0.00	0.0	-	-
500.0	87.30	27.72	17.46	5.54	0.00	5.10	0.00	1.02
750.0	N/A	259.91	N/A	34.65	N/A	35.10	N/A	4.68
1000.0	534.90	384.60	53.49	38.46	57.57	94.30	5.75	9.43
1250.0	N/A	554.72	N/A	44.37	N/A	247.60	N/A	19.80
1750.0	1992.11	969.29	113.83	55.38	572.93	543.80	32.73	31.17
2500.0	2727.55	1774.48	109.10	70.97	808.00	1540.40	32.32	61.61
5000.0	5145.48	4344.10	102.90	86.88	829.40	4065.20	16.58	81.30

N/A (not applicable) indicates concentrations that were intentionally not tested as part of the experimental design

### Lethal endpoint

#### Mortality

At the end of the test, control mortality was 6% for La and 3.8% for Gd. After 1 day of exposure, the mortality reached around 14% for Gd and 17% for La at the highest concentration; therefore, only the LC_10_ could be obtained. Moreover, after 2 days of exposure, the mortality of La reached 51% on average; therefore, an accurate LC_50_ within the tested concentrations could not be estimated. The daily LC_50_, LC_20_, and LC_10_ values were calculated with measured concentrations at *t* = 7 and are presented in Table 2.

**Table 2:** Lethal measured concentration and standard error (µg L^−1^) causing 50%, 20%, and 10% mortality (LC_50_, LC_20_, LC_10_) of La and Gd for every day of exposure

REE	Day	LC_50_ (µg L^−1^)	Standard error	LC_20_ (µg L^−1^)	Standard error	LC_10_ (µg L^−1^)	Standard error
La	1	-	-	-	-	2959.54	464.32
	2	-	-	602.89	197.78	98.09	61.61
	3	2561.06	1114.71	450.46	89.85	162.99	65.37
	4	783.98	18.84	637.48	28.01	564.82	36.19
	5	707.31	91.96	151.85	37.73	61.74	23.55
	6	495.33	64.06	90.90	24.88	33.72	13.58
	7	403.67	50.68	78.96	21.34	30.40	11.77
Gd	1	**-**	-	**-**	-	2997.67	642.01
	2	2923.26	635.81	416.55	69.71	133.25	31.00
	3	989.27	127.44	198.29	30.15	77.45	16.20
	4	573.45	89.70	87.13	17.15	28.94	7.81
	5	343.86	45.50	50.34	9.58	16.36	4.29
	6	287.78	34.09	45.52	8.17	15.48	3.80
	7	241.28	29.13	33.73	6.38	10.67	2.77

The values obtained for all percentages of lethality, and for both metals, declined with exposure time (except La day 3 and 4 of LC_20_ and LC_10_), as the mortality continued increasing during the exposure and values remained consistently higher for La rather than for Gd.

### Sublethal endpoints

#### Feeding rate

**Fig. 1:**
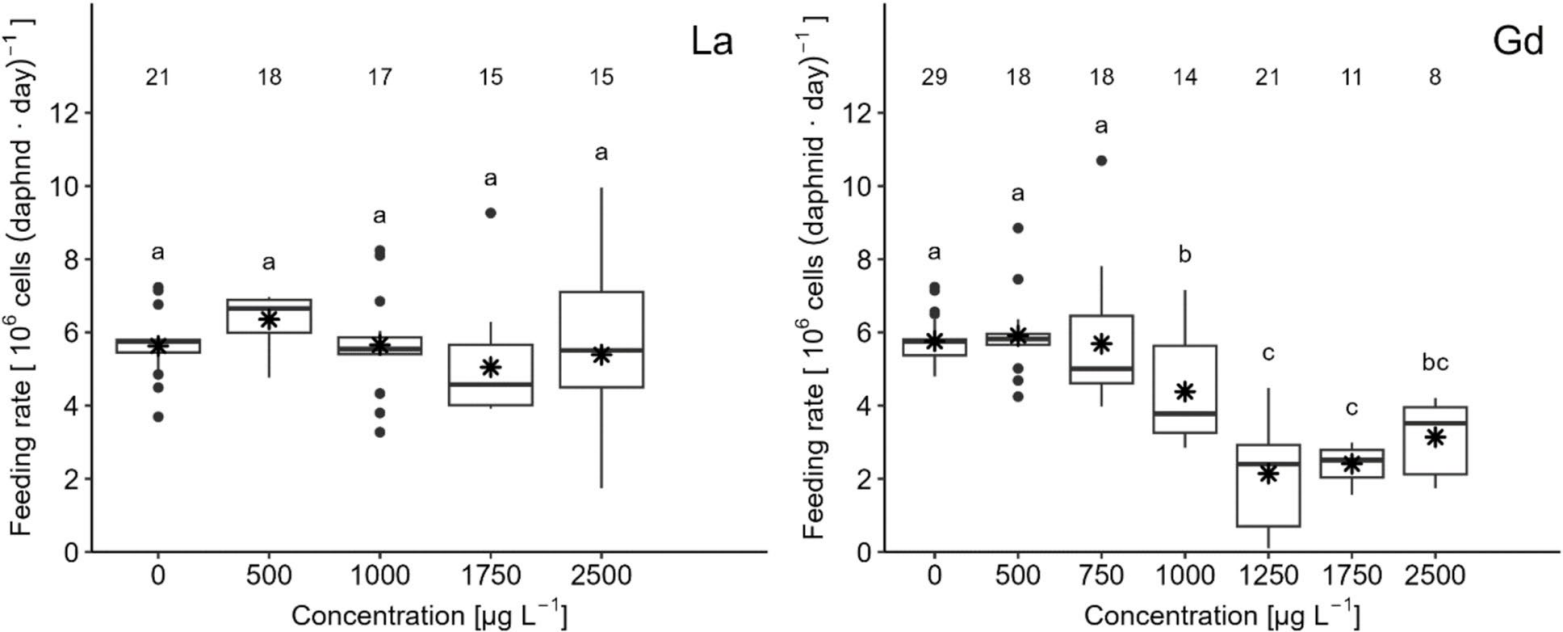
Boxplot illustrating the feeding rate (cells consumed per daphnid per day) of *D. magna* exposed to La and Gd. Letters indicate significant differences between treatment groups (*p* > 0.05), while asterisks denote the mean values. The number of daphnia in each treatment is provided above the box plots

The mean values of the feeding rate (*R. subcapitata* cells consumed by daphnid per day) for the 7 days of exposure are shown in Fig. 1. Under control conditions, the feeding rate was around 6 × 10^6^ cells consumed per daphnid per day.

For the La exposure, the feeding rate was not inhibited by the lowest concentrations of La, 500, and 1000 µg L^−1^, and the rate increased by 14 and 8%, respectively, in comparison to the control. The concentration of 1750 µg L^−1^ caused the highest decrease, with an average of 5 × 10^6^ cells consumed per daphnid per day, which represents about 14% less compared to the control. Moreover, none of the concentrations caused a significant change in the feeding rate compared to the control or between treatments (*p*-values provided in SI).

For the Gd exposure, the lowest concentration, 500 µg L^−1^, did not affect the feeding rate, and only from the second lowest concentration, 750 µg L^−1^, a reduction could be observed. The nominal concentration of 1000 µg L^−1^ caused a reduction in the feeding rate by approximately 26% compared to the control. From this concentration onwards, the feeding rate was significantly reduced (*p*-values provided in SI). At 1250 µg L^−1^, the highest reduction of the feeding rate occurred at about 65%, where 2.1 × 10^6^ cells per daphnid per day were consumed. A slight increase in feeding was observed at concentrations 1750 and 2500 µg L^−1^, where the reduction in feeding rate was approximately 60 and 47%, respectively.

The EC_10_ and EC_20_ values obtained for the feeding rate inhibition are presented in Table 3.

**Table 3:** Measured concentrations (µg L^−1^) of La and Gd causing 10 and 20% of effects on the feeding rate, somatic growth, and maturity of *D. magna* with their corresponding standard errors

REE	Endpoint	EC_20_ (µg L^−1^)	Standard error	EC_10_ (µg L^−1^)	Standard error
La	Feeding	124.86	233.38	95.32	194.41
	Somatic growth	287.60	706.35	61.19	151.53
	Maturity	0.79	1.09	0.26	0.43
Gd	Feeding	68.67	18.28	52.82	23.62
	Somatic growth	97.93	37.80	44.00	15.57
	Maturity	0.39	0.18	0.14	0.08

#### Somatic growth

**Fig. 2:**
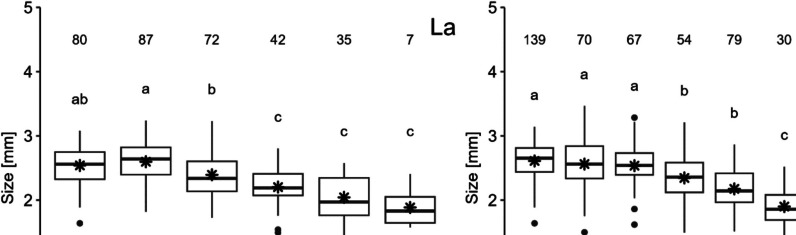
Boxplot illustrating the size of *D. magna* at the end of exposure (*t* = 7 days) to La and Gd. Letters indicate significant differences between treatment groups (*p* > 0.05), while asterisks denote the mean values. The number of daphnia in each treatment is provided above the box plots

The mean length (mm) of the daphnids after 7 days of exposure is shown in Fig. 2. Under control conditions, the mean length was about 2.5 mm, and this size decreased with increasing concentration.

In the exposure to La, the lowest concentration, the length of the daphnids was on average 2.60 mm, which was about 2% larger than the control organisms. The following concentration of 1000 µg L^−1^ caused a significant reduction in comparison to the control (*p* = 0.03); an average length of 2.39 mm was measured at this concentration. This significant size reduction was observed in the remaining higher concentrations (*p*-values provided in SI). The highest concentration of exposure 5000 µg L^−1^ caused the highest reduction of growth, with a length of 1.89 mm on average, which is about a 26% reduction in comparison to the control.

As for the Gd exposure, all the concentrations of exposure caused a reduction of the daphnid length. A significant reduction was caused from 1000 µg L^−1^ (*p* < 0.001) onwards (further *p*-values provided in SI). The highest reduction was observed at the second highest concentration of exposure (2500 µg L^−1^) with an average size of 1.71 mm, corresponding to a 30% reduction compared to the control.

The EC_10_ and EC_20_ obtained for the growth inhibition are presented in Table 3.

#### Relationship between feeding and size

**Fig. 3:**
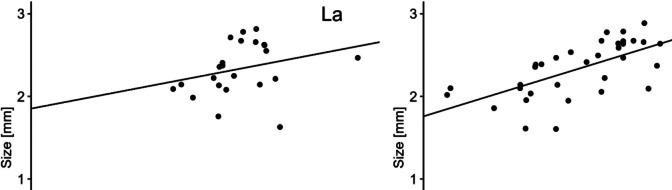
Linear regression of the feeding rate (cells consumed per daphnid per day) and size (mm) of *Daphnia magna* exposed to La (*n* = 23) and Gd (*n* = 36)

The strength of the relationship between growth and feeding rate was assessed by linear regression (Fig. 3). The correlation for both La and Gd had a positive slope, indicating a positive relationship between the two measurements. Additionally, the *R*^2^ for La and Gd was estimated as 0.03 and 0.42, respectively, indicating that the measurements for La have little to no correlation, while Gd had a stronger correlation. However, the difference between the correlations is also affected by the number of samples tested for La (*n* = 23) and for Gd (*n* = 36).

#### Maturity

**Fig. 4:**
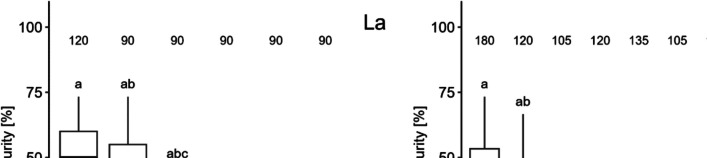
Boxplot illustrating the percentage of maturity of *D. magna* exposed to La and Gd. Letters indicate significant differences between treatment groups (*p* > 0.05), while asterisks denote the mean values. The number of daphnia in each treatment is provided above the box plots

Approximately 40% of the control population matured after 7 days (Fig. 4). Exposure to La and Gd significantly decreased the number of animals maturing, in a dose-dependent way. However, Gd caused a higher inhibitory effect than La. At the end of the experiment, no daphnid exposed to nominal concentrations of 2500 µg L^−1^ for La and 1750 µg L^−1^ for Gd or higher could reach maturity.

The EC_10_ and EC_20_ obtained for the maturity inhibition are presented in Table 3.

## Discussion

### Influence of bioavailability on toxicity

In this study, the dissolved concentration rather than the total concentration present in the media was estimated by the filtration of the solutions before the analysis. The initial dissolved concentration was greatly reduced during the 7 days. The discrepancy between the dissolved concentrations at the start of the test and the progressive decrease during the exposure has already been observed in *Daphnia* tests of 48-h exposure (Lachaux et al. 2022; Romero-Freire et al. 2019; Vukov et al. 2016) and 21-day exposure (Blinova et al. 2022).

The media composition is one of the main factors influencing the availability of lanthanoids (Romero-Freire et al. 2019). Properties such as the water hardness, alkalinity, and dissolved organic carbon will influence the formation of chemical ligands, which can cause lanthanoids to form complexes and potentially precipitate, decreasing the fraction that is available to interact with the organisms (Herrmann et al. 2016; Romero-Freire et al. 2019). However, Blinova et al. (2022) and Revel et al. (2023) suggest that another route of exposure for the daphnids is by the ingestion of precipitated insoluble or partially soluble particulate fractions. In the exposure method employed in this current study, along with the complexation, the available fraction might have also been influenced by the presence of algae as a food source and the static conditions of the test. Vasconcelos and Leal (2008) described that algae exudates can increase the binding of trace metals on algae. Therefore, it could be expected that during the exposure, daphnids were further exposed by the ingestion of lanthanoids bound to the algae.

Under the study’s exposure conditions, Gd maintained a greater soluble fraction and higher toxicity compared to La. While the highest concentrations of La decreased from 70 to 80%, Gd could maintain its dissolved fraction with decreases of about 10% at the highest concentrations. Borgmann et al. (2005) from an extensive metal toxicity study determined that the more toxic metals had higher solubility. By the end of the test, the recovery rates of Gd classify it into the soluble group (> 75% recovery), while La is classified as partly soluble (10–75% recovery). Moreover, it is also important to notice that this classification accounts for only the highest concentrations of exposure of this test (> 1250 µg L^−1^ nominal), as in the lower concentrations, both elements would be considered as sparingly soluble (< 10% recovery).

The behavior of individual REEs in terms of speciation and adsorption is important for a better understanding of toxicodynamic processes. The study of Kang et al. (2022) assessed the toxicity of multiple lanthanoids, specifically two light (La and Ce) and two heavy (Gd and Ho), on zebrafish (*Danio rerio)* at different pH levels, carbonate, and sulfate contents. Authors observed no differences of toxicity at conditions that allow higher soluble forms, suggesting the potential toxicity is influenced by the bioavailability rather than the inherent toxicity of the elements themselves. So far, REEs (specially lanthanoids) are commonly studied as a group due to their similar chemical properties; however, when assessing the toxicity, there is still no clear pattern observed even if similar exposure conditions are used. Studies reveal either increasing toxicity with increasing atomic number (González et al. 2015; Weltje et al. 2002b), decreased toxicity with increasing atomic number (Kang et al. 2022), or very little influence (Lachaux et al. 2022). Therefore, while bioavailability plays a significant role in toxicity, our physicochemical understanding of REEs needs to improve further to unravel the factors influencing the toxicodynamic and toxicokinetic processes of individual REEs. In this study, we observed that the inherent properties of Gd contribute to its higher toxicity compared to La, highlighting the complex interplay between bioavailability and toxicity for these specific lanthanoids, while the behaviors of other lanthanoids remain uncertain.

### Toxicity endpoints

The exposure to La and Gd induced adverse effects on the daphnids, as well as in the algae provided as food source. During the exposure period, algae agglomerations, more prominent at higher concentrations (starting from nominal exposure of 1000 µg L^−1^) were observed. Similar behavior was observed in studies of 72-h exposure of unicellular algae (Bergsten-Torralba et al. 2020; Romero-Freire et al. 2019). A visual representation of the agglomerates in this study is provided in the Supplementary Information. Furthermore, the formation of agglomerations might have influenced the amount of food available, as *Daphnia* have the ability to ingest particles up to 50 µm in size (Geller and Müller 1981).

The La exposure induced subtle changes in feeding behavior of daphnids. Specifically, there was an increased feeding at lower concentrations and a decrease at higher concentrations. However, these changes did not reach statistical significance. For the Gd exposure, a constant decrease was observed at all concentrations of exposure, with significant decrease at concentration higher than 1000 µg L^−1^. The variations in feeding behavior suggest that the formation of algae agglomerates might not be the primary cause of the decrease in daphnid feeding. Despite the formation of agglomerates in the exposures of both elements, the effects on feeding behavior were influenced differently. Consequently, the reduced feeding rate could be more closely associated with the damage caused by exposure to the soluble fraction of lanthanoids. Moreover, the exposure also induced a decrease in the somatic growth of the daphnids, both elements causing a significant reduction in size with increasing concentration. Even though the size measurements at higher concentrations were limited due to the reduced number of alive daphnids, a clear trend can be observed with the obtained mean values.

It has been widely studied that the growth and development of daphnids is heavily influenced by their feeding (Green 1956; Ingle et al. 1937). Consequently, this study included an analysis of the correlation between the feeding rate (cells consumed per daphnid per day) and the size (mm). This aimed to enhance the understanding of the relationship between these two endpoints. Results indicate that there is not a strong correlation of both variables in either La or Gd exposure. However, a limiting factor in this study is that for both endpoints, the analysis was carried out at the end of the test, accounting for algae concentration and alive daphnids. By considering only alive daphnids, the size of the daphnids at the time of death is neglected, limiting the study of their algae consumption while alive, specially at higher concentrations with high mortality rates. Moreover, the study of this link could be further improved if the algae concentration was measured daily.

Furthermore, the effects on the growth could be explained by the potential of lanthanoid ions (Ln^3+^) to mimic or block the function of Ca^2+^ channels in cell membranes (Das et al. 1988). Earlier studies described that this might occur because Ln^3+^ have a much higher charge-to-volume ratio compared to that of Ca^2+^; therefore, they have higher affinity to binding sites (Evans 1983). In *Daphnia* species, calcium is required for the calcification of their exoskeleton, a process that is highly linked to their moulting (Porcella et al. 1969), therefore also strongly associated to their development and changing of instars. This replacement might be more detrimental for younger daphnids, who have a higher requirement of Ca to frequently moult and grow and still maintain the adequate levels of Ca to maintain intracellular calcium homeostasis before calcification (Hessen and Alstad Rukke 2000). At the start of the test, daphnids exposed were less than 24 h of age, therefore going through a developmental phase during the exposure period. It can be assumed that if the lanthanoids were disrupting the calcium uptake, daphnids exposed were potentially having longer duration of instars, which consequentially led to smaller organisms. Additionally, exposure to REEs can initiate detoxification processes in *Daphnia*, diverting their energy reserves towards detoxification rather than allocating them to essential processes like growth and reproduction (Egler et al. 2023; Galdiero et al. 2019).

As for the reproduction endpoints, considered as the maturity, it was the most sensitive endpoint measured on the test for both La and Gd, with the latter being the most toxic among both. From early daphnid studies, it was observed that the egg production was inherently related to growth, therefore affected if growth was disrupted (Green 1956). Nevertheless, it is important to highlight that even though the longer exposure allows for additional endpoints compared to acute tests, 7 days is still a short period of time to fully evaluate the effects caused on the reproduction.

Moreover, Gd exhibited a greater soluble fraction compared to La and demonstrated higher toxicity across all endpoints measured. This increased toxicity in Gd may be attributed to its higher soluble fraction, leading to more direct contact with the organisms, which could affect development through mechanisms such as altered physiological responses or interference with metabolic processes. In contrast, La’s lower solubility may limit its bioavailability, potentially resulting in different developmental impacts. Additionally, while exposure through the ingestion of insoluble fractions, such as those bound to algae, may occur, this study emphasizes the significance of direct exposure to soluble Gd. It is important to note that although this study focuses on Gd and La, the observed toxicity may also reflect characteristics common to REEs in general, highlighting the need for further research to clarify the specific mechanisms involved.

### Variability of toxicity due to test conditions

Data about multiple days of exposure are available for both La and Gd, ranging from 14-day (Lürling and Tolman 2010) to 31-day test (Blinova et al. 2022) and including the standard exposure of 21 days (Barry and Meehan 2000; Blinova et al. 2018; Salvatore et al. 2024).

Blinova et al. (2018) calculated the 21-day nominal LC_50_ for *D. magna* as 490 µg L^−1^ for Gd(NO_3_)_3_ and 460 µg L^−1^ for La(NO_3_)_3_. The closest values in our study correspond to the 4-day LC_50_ of Gd (573.45 µg L^−1^) and the 6-day LC_50_ of La (495.33 µg L^−1^). However, It is important to note that the values reported in our study were based on measured filtrated concentrations, which generally yield lower estimates compared to nominal concentrations.

Lürling and Tolman (2010) found that in media without or with phosphate at a concentration of 330 µg L^−1^, measured concentrations of La up to 1000 µg L^−1^ did not significantly affect mortality or the age of first reproduction in a 14-day exposure period of *D. magna* to La(NO_3_)_3_. In contrast, in our study, values below 1000 µg L^−1^ were obtained as LC_50_ from days four for La (783.98 µg L^−1^) and 3 days of exposure for Gd (989.27 µg L^−1^), displaying a higher toxicity regarding mortality. Moreover, Lürling and Tolman (2010) further observed that in the presence of phosphate, the growth and size at first growth were notably reduced at concentrations starting from 94.1 µg L^−1^. In our study, however, no significant effects were observed at the lowest tested nominal concentration of 500 µg L^−1^ for the sublethal endpoints measured.

In the study by Barry and Meehan (2000), which investigated the effects of prolonged exposures of LaCl_3_ on *D. carinata*, measured concentrations of up to 40 µg L^−1^ had no significant impact on mortality, while concentrations up to 30 µg L^−1^ did not significantly affect reproduction after 21 days of exposure. However, when the total hardness was elevated from 98 to 160 mgL^−1^ CaCO_3_, the mortality rate reached 100% after 4 days at concentrations of 49 µg L^−1^. In contrast, our study, conducted in M4 Elendt media with a hardness of approximately 100–200 mg/L as CaCO₃, observed a significantly higher 7-day LC50, showing a one-order-of-magnitude difference. Notably, the 7-day LC_10_ of La (30.40 µg L⁻^1^) and the 7-day LC_20_ of Gd (33.73 µg L⁻^1^) for induced mortality are closest to these findings.

Furthermore, Blinova et al. (2022) performed a 31-day test on *D. magna* exposed to a nominal concentration of 100 µg L^−1^ of Gd(NO_3_)_3_, with measured concentrations ranging from 82 to 77 µg L^−1^. The authors did not observe effects on mortality, growth, or reproduction endpoints. In contrast, our study demonstrated that lower concentrations of La and Gd induced notable effects after just 7 days. Specifically, we obtained 7-day LC_10_ values of 30.40 µg L⁻^1^ for La and 10.67 µg L⁻^1^ for Gd. Moreover, we found LC_20_ values of 78.96 µg L⁻^1^ for La and 33.73 µg L⁻^1^ for Gd. Additionally, our effective concentrations indicated that even lower concentrations caused significant sublethal effects.

In the study of Salvatore et al. (2024), a 21-day exposure to yttrium (as YCl_3_ × 7 H_2_O) in *Daphnia magna* demonstrated that a concentration of 27 µg L⁻^1^ induced a significant mortality from day 14, reaching 16% decreased of the treated group by the end of the test. In our 7-day test, we found that similar concentrations were estimated for lethal concentrations at the end of the test, with an LC_20_ value of 33 µg L⁻^1^ for Gd and an LC_10_ value of 30.40 µg L⁻^1^ for La. Furthermore, Salvatore et al. (2024) reported delayed maturity and reduced clutch size, as well as changes in the metabolomics of *Daphnia magna*.

While these studies assessed multiple endpoints related to mortality, reproduction, and growth, the difficulty in the comparison of their results arises from variations in the statistical endpoints employed to present their findings. Furthermore, variations in the composition of the media are important factors contributing to differences in the toxicity observed in tests conducted under similar conditions. Higher differences between results can be observed when testing conditions are altered, such as the duration of exposure or the chemical form of the REE.

### Relevance of test approach methodology

The results obtained in this study indicate that it is possible to assess multiple endpoints in daphnids exposed during 7 days and to express results in effect concentrations. However, due to the static conditions and the presence of a food source, more accurate results might be obtained for substances that have a higher solubility and can be maintained in solution during the exposure period, such as the case for Gd compared to La. In addition, a better understanding of the speciation of the exposure chemicals would be necessary to obtain further information on the bioavailability.

The test conditions employed in this study serve as a viable alternative to provide a comprehensive preliminary overview of lethal and sublethal effects. While not a complete substitute to the reproduction standard test, it could be used as alternative to range finding tests. As previously noted by Adams and Heidolph (1985), Lewis and Horning (1988), and Winner (1988), shorter 7-day tests can yield results comparable to those from longer exposure periods. However, it is essential to adapt testing protocols to the specific chemicals being evaluated to accurately extrapolate the findings to relevant ecological contexts. Moreover, exposure conditions can be further adjusted to align with the objectives of subsequent studies. For instance, modifications may include changes in the number of exposed daphnids per replicate, the amount of food provided, and the frequency of test medium renewal. Additional evaluations could also encompass endpoints related to daphnid physical fitness, behavior, and assessments of food quality based on the algae used as a food source.

One of the substantial differences between the exposure conducted in this work and the acute standard test is the addition of food. On one hand, a food source is crucial to maintain low mortality in the control conditions during the longer exposure, yet it can also play an important role in the apparent toxicity observed. For instance, food availability affects the sensitivity of the daphnids as they do not experience starvation during the exposure (Biesinger and Christensen 1972), changing the worst-case scenario principle. Furthermore, in this study, the conditions were kept static, differing from the reproduction test guideline that mandates a maximum of 3 days for medium renewal. A constant renewal is particularly suggested for substances with recoveries below 80%, which was the case for lower La concentrations. Although higher time consumption would be required for the preparation of solutions and media exchange, considering a media exchange might be specially relevant for LREEs.

The results indicate that lethal concentrations exhibited decreasing standard errors with increasing exposure days, indicating improved reliability of the data over time. This trend may suggest that longer exposure periods allow for a more accurate assessment of the toxic effects of both lanthanum (La) and gadolinium (Gd) on *Daphnia magna*. Notably, while mortality endpoints provided crucial insights into acute toxicity, the sublethal endpoints showed more pronounced responses with comparatively lower EC values. This discrepancy highlights the necessity of assessing both lethal and sublethal effects in toxicity evaluations, as sublethal endpoints can uncover subtle effects that may be missed when relying solely on acute mortality assessments. The consistent patterns observed across different exposure durations reinforce the notion that longer-term studies can yield more insights into our understandings of the ecological implications of REE exposure.

However, a potential challenge that could arise from the use of alternative testing methods is the variations of the experimental conditions, limiting the comparison between studies. This issue is already observed for REEs tested under standardized conditions. The presence of large quantities of data that are incompatible for comparison may hinder the establishment of threshold limits for environmental safety. Nevertheless, to address this issue, it might be more relevant to clearly report of all relevant test conditions rather than try to maintain standardized conditions.

## Conclusion

In this study, the effects of La and Gd on *Daphna magna* were evaluated under a 7-day exposure period. Effective concentrations were estimated for the endpoints of mortality, somatic growth, feeding rate, and maturity. Results obtained showed that maturity was the most sensitive endpoint evaluated. Moreover, Gd induced higher toxicity in comparison to La in all the endpoints, which could be highly linked to the ability to stay in solution during the exposure period.

Furthermore, the test approach methodology can provide a comprehensive overview of effects over longer exposure periods compared to acute methods. It could also serve as an early screening tool for identifying a range of concentrations and effects that might be relevant for the standardized reproduction test method.

## Supplementary information

Below is the link to the electronic supplementary material.

Supplementary file1 (490 KB)

## Data Availability

The authors declare that the data supporting the findings of this study are available within the paper and its Supplementary Information files. Should any raw data files be needed in another format, they are available from the corresponding author upon reasonable request.
