# Supplementary material for: Sublethal and lethal toxicity assessment of lanthanum and gadolinium to Daphnia magna in a 7-day test method
Source: Environ Sci Pollut Res Int. 2025 Jan 10;32(5):2467–78. doi: 10.1007/s11356-024-35854-7 (PMC11802601; doi:10.1007/s11356-024-35854-7)
Supplement: Supplementary file 1 — Supplementary file1 (490 KB) [file 11356_2024_35854_MOESM1_ESM.docx]

**Toxicity assessment of Lanthanum and Gadolinium to *Daphnia magna* in a seven-day test method**

**Supplementary data**

Table 1 Growth constants of Raphidocelis subcapitata under the 7-day test conditions used for Daphnia magna.

| **Concentration [µgL^-1^]** | **La** | **Gd** |
| --- | --- | --- |
| 0 | 0.33 | 0.33 |
| 500 | 0.38 | 0.33 |
| 750 | - | 0.31 |
| 1000 | 0.38 | 0.24 |
| 1250 | - | 0.21 |
| 1750 | 0.33 | 0.17 |
| 2500 | 0.35 | 0.21 |
| 5000 | 0.32 | 0.18 |

Table 2 Tukey’s test comparison of D. magna feeding after 7 days of exposure to various La and Gd concentrations. Significance levels: p < 0.001, p < 0.01, p < 0.05, p < 0.1, and p < 1 (not significant) are indicated as follows: ‘***’, ‘**’, ‘*’, ‘·’, and ‘/’.

| **Element** | **Group** | **Mean difference** | **Std. Error** | **p value** | **Significance level** |
| --- | --- | --- | --- | --- | --- |
| La | 500 – 0 | 728303 | 525298 | 0.637 | / |
|  | 1000 - 0 | 511613 | 525298 | 0.866 | / |
|  | 1750 - 0 | -579499 | 552860 | 0.832 | / |
|  | 2500 – 0 | -240431 | 552860 | 0.992 | / |
|  | 1000 – 500 | -216690 | 545127 | 0.995 | / |
|  | 1750 - 500 | -1307801 | 571734 | 0.159 | / |
|  | 2500 – 500 | -968734 | 571734 | 0.442 | / |
|  | 1750 - 1000 | -1091111 | 571734 | 0.321 | / |
|  | 2500 – 1000 | -752043 | 571734 | 0.682 | / |
|  | 2500 - 1750 | 339068 | 597157 | 0.979 | / |
|  |  |  |  |  |  |
| Gd | 500 - 0 | 151876 | 348613 | 0.999 | / |
|  | 750 – 0 | -69805 | 348613 | 1.000 | / |
|  | 1000 – 0 | -1377374 | 378095 | 0.007 | ** |
|  | 1250 – 0 | -3618895 | 332894 | < 0.001 | *** |
|  | 1750 – 0 | -3353968 | 411401 | < 0.001 | *** |
|  | 2500 – 0 | -2622951 | 463967 | < 0.001 | *** |
|  | 7500 – 500 | -221681 | 387266 | 0.997 | / |
|  | 1000 – 500 | -1529250 | 414004 | 0.006 | ** |
|  | 1250 – 500 | -3770771 | 373178 | < 0.001 | *** |
|  | 1750 – 500 | -3505844 | 444628 | < 0.001 | *** |
|  | 2500 – 500 | -2774826 | 493669 | < 0.001 | *** |
|  | 1000 – 750 | -1307569 | 414004 | 0.031 | * |
|  | 1250 – 750 | -3549090 | 373178 | < 0.001 | *** |
|  | 1750 – 750 | -3284164 | 444628 | < 0.001 | *** |
|  | 2500 - 750 | -2553146 | 493669 | < 0.001 | *** |
|  | 1250 – 1000 | -2241521 | 400858 | < 0.001 | *** |
|  | 1750 – 1000 | -1976595 | 468101 | < 0.001 | *** |
|  | 2500 – 1000 | -1245577 | 514911 | 0.195 | / |
|  | 1750 – 1250 | 264926 | 432413 | 0.996 | / |
|  | 2500 – 1250 | 995944 | 482697 | 0.375 | / |
|  | 2500 – 1750 | 731018 | 539841 | 0.820 | / |

Table 3 Tukey’s test comparison of D. magna size after 7 days of exposure to various La and Gd concentrations. Significance levels: p < 0.001, p < 0.01, p < 0.05, p < 0.1, and p < 1 (not significant) are indicated as follows: ‘***’, ‘**’, ‘*’, ‘·’, and ‘/’.

| **Element** | **Group** | **Mean difference** | **Std. Error** | **p value** | **Significance level** |
| --- | --- | --- | --- | --- | --- |
| La | 500 – 0 | 0.05788 | 0.04888 | 0.832 | / |
|  | 1000 – 0 | -0.14467 | 0.05127 | 0.051 | · |
|  | 1750 – 0 | -0.33679 | 0.06014 | <0.001 | *** |
|  | 2500 – 0 | -0.49641 | 0.06396 | <0.001 | *** |
|  | 1000 – 500 | -0.65355 | 0.12439 | <0.001 | *** |
|  | 1750 – 500 | -0.20255 | 0.05028 | <0.001 | *** |
|  | 2500 – 500 | -0.39466 | 0.0593 | <0.001 | *** |
|  | 1750 – 1000 | -0.55428 | 0.06317 | <0.001 | *** |
|  | 2500 – 1000 | -0.71143 | 0.12399 | <0.001 | *** |
|  | 2500 – 1750 | -0.19211 | 0.06127 | 0.020 | * |
|  |  |  |  |  |  |
| Gd | 500 – 0 | -0.048 | 0.050 | 0.956 | / |
|  | 750 – 0 | -0.074 | 0.051 | 0.756 | / |
|  | 1000 – 0 | -0.265 | 0.055 | < 0.001 | *** |
|  | 1250 – 0 | -0.435 | 0.048 | < 0.001 | *** |
|  | 1750 – 0 | -0.710 | 0.069 | < 0.001 | *** |
|  | 2500 – 0 | -0.892 | 0.143 | < 0.001 | *** |
|  | 750 – 500 | -0.025 | 0.059 | 0.999 | / |
|  | 1000 – 500 | -0.216 | 0.062 | 0.008 | ** |
|  | 1250 – 500 | -0.386 | 0.056 | < 0.001 | *** |
|  | 1750 – 500 | -0.662 | 0.075 | < 0.001 | *** |
|  | 2500 – 500 | -0.843 | 0.146 | < 0.001 | *** |
|  | 1000 – 750 | -0.191 | 0.063 | 0.034 | * |
|  | 1250 – 750 | -0.361 | 0.057 | < 0.001 | *** |
|  | 1750 – 750 | -0.636 | 0.075 | < 0.001 | *** |
|  | 2500 – 750 | -0.818 | 0.146 | < 0.001 | *** |
|  | 1250 – 1000 | -0.170 | 0.061 | 0.067 | · |
|  | 1750 – 1000 | -0.445 | 0.078 | < 0.001 | *** |
|  | 2500 – 1000 | -0.627 | 0.147 | < 0.001 | *** |
|  | 1750 – 1250 | -0.275 | 0.074 | 0.003 | ** |
|  | 2500 – 1250 | -0.457 | 0.145 | 0.025 | * |
|  | 2500 – 1750 | -0.182 | 0.153 | 0.889 | / |

Table 4 Tukey’s test comparison of D. magna maturity after 7 days of exposure to various La and Gd concentrations. Significance levels: p < 0.001, p < 0.01, p < 0.05, p < 0.1, and p < 1 (not significant) are indicated as follows: ‘***’, ‘**’, ‘*’, ‘·’, and ‘/’.

|  | **Linear Hypotheses** | **Estimate** | **Std. Error** | **p value** | **Significance level** |
| --- | --- | --- | --- | --- | --- |
| La | 500 – 0 | -5.83E+00 | 1.01E+01 | 0.992 | / |
|  | 1000 – 0 | -2.81E+01 | 1.01E+01 | 0.089 | · |
|  | 1750 – 0 | -3.36E+01 | 1.01E+01 | 0.025 | * |
|  | 2500 – 0 | -4.25E+01 | 1.01E+01 | 0.003 | ** |
|  | 500 – 0 | -4.25E+01 | 1.01E+01 | 0.003 | ** |
|  | 1000 – 500 | -2.22E+01 | 1.08E+01 | 0.337 | / |
|  | 1750 – 500 | -2.78E+01 | 1.08E+01 | 0.136 | / |
|  | 2500 – 500 | -3.67E+01 | 1.08E+01 | 0.021 | * |
|  | 5000 – 500 | -3.67E+01 | 1.08E+01 | 0.021 | * |
|  | 1750 – 1000 | -5.56E+00 | 1.08E+01 | 0.995 | / |
|  | 2500 – 1000 | -1.44E+01 | 1.08E+01 | 0.764 | / |
|  | 5000 – 1000 | -1.44E+01 | 1.08E+01 | 0.764 | / |
|  | 2500 – 1750 | -8.89E+00 | 1.08E+01 | 0.961 | / |
|  | 5000 – 1750 | -8.89E+00 | 1.08E+01 | 0.961 | / |
|  | 5000 – 2500 | 2.13E-14 | 1.08E+01 | 1.000 | / |
|  |  |  |  |  |  |
| Gd | 500 – 0 | -8.06E+00 | 5.61E+00 | 0.836 | / |
|  | 750 – 0 | -2.27E+01 | 5.85E+00 | 0.006 | ** |
|  | 1000 – 0 | -3.39E+01 | 5.61E+00 | < 0.001 | *** |
|  | 1250 – 0 | -3.67E+01 | 5.42E+00 | < 0.001 | *** |
|  | 1750 – 0 | -3.89E+01 | 5.85E+00 | < 0.001 | *** |
|  | 2500 – 0 | -3.89E+01 | 5.26E+00 | < 0.001 | *** |
|  | 5000 – 0 | -3.89E+01 | 5.42E+00 | < 0.001 | *** |
|  | 750 – 500 | -1.46E+01 | 6.36E+00 | 0.308 | / |
|  | 1000 – 500 | -2.58E+01 | 6.15E+00 | 0.002 | ** |
|  | 1250 – 500 | -2.86E+01 | 5.97E+00 | < 0.001 | *** |
|  | 1750 – 500 | -3.08E+01 | 6.36E+00 | < 0.001 | *** |
|  | 2500 – 500 | -3.08E+01 | 5.83E+00 | < 0.001 | *** |
|  | 5000 – 500 | -3.08E+01 | 5.97E+00 | < 0.001 | *** |
|  | 1000 – 750 | -1.12E+01 | 6.36E+00 | 0.647 | / |
|  | 1250 – 750 | -1.40E+01 | 6.19E+00 | 0.333 | / |
|  | 1750 – 750 | -1.62E+01 | 6.57E+00 | 0.229 | / |
|  | 2500 – 750 | -1.62E+01 | 6.06E+00 | 0.150 | / |
|  | 5000 – 750 | -1.62E+01 | 6.19E+00 | 0.170 | / |
|  | 1250 – 1000 | -2.78E+00 | 5.97E+00 | 1.000 | / |
|  | 1750 – 1000 | -5.00E+00 | 6.36E+00 | 0.993 | / |
|  | 2500 – 1000 | -5.00E+00 | 5.83E+00 | 0.989 | / |
|  | 5000 – 1000 | -5.00E+00 | 5.97E+00 | 0.990 | / |
|  | 1750 – 1250 | -2.22E+00 | 6.19E+00 | 1.000 | / |
|  | 2500 – 1250 | -2.22E+00 | 5.65E+00 | 1.000 | / |
|  | 5000 – 1250 | -2.22E+00 | 5.79E+00 | 1.000 | / |
|  | 2500 – 1750 | 6.40E-14 | 6.06E+00 | 1.000 | / |
|  | 5000 – 1750 | 2.13E-14 | 6.19E+00 | 1.000 | / |
|  | 5000 - 2500 | -4.26E-14 | 5.65E+00 | 1.000 | / |


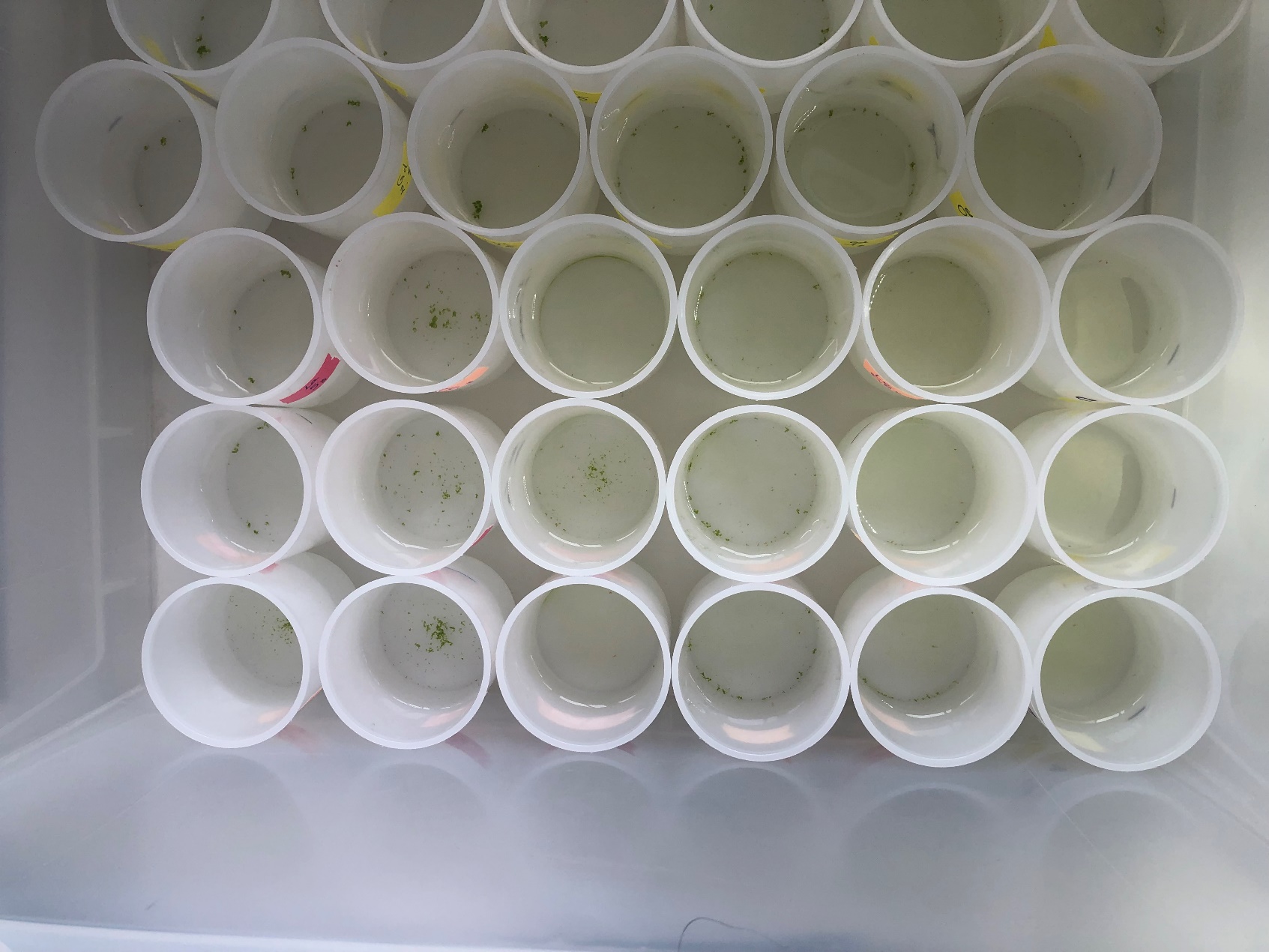


Figure 1. Graphical display of algae agglomerates formed after a 7-day exposure to La. The concentration arrangement follows a left-to-right progression, ranging from the control (0.00 µgL^-1^) to the highest concentration (5000 µgL^-1^). Within each concentration, there are three replicates, organized vertically.
